# Symbiosis in the microbial world: from ecology to genome evolution

**DOI:** 10.1242/bio.032524

**Published:** 2018-02-15

**Authors:** Jean-Baptiste Raina, Laura Eme, F. Joseph Pollock, Anja Spang, John M. Archibald, Tom A. Williams

**Affiliations:** 1 Climate Change Cluster, University of Technology Sydney, Ultimo, NSW 2007, Australia; 2 Department of Cell and Molecular Biology, Science for Life Laboratory, Uppsala University, SE-75123, Uppsala, Sweden; 3 Eberly College of Science, Department of Biology, Pennsylvania State University, University Park, PA 16801, USA; 4 NIOZ, Royal Netherlands Institute for Sea Research, Department of Marine Microbiology and Biogeochemistry, and Utrecht University, P.O. Box 59, NL-1790 AB Den Burg, The Netherlands; 5 Department of Biochemistry & Molecular Biology, Dalhousie University, Halifax, NS, B3H 4R2, Canada; 6 School of Biological Sciences, University of Bristol, 24 Tyndall Ave, Bristol, BS8 1TH, UK

**Keywords:** Ecology, Evolution, Symbiosis

## Abstract

The concept of symbiosis – defined in 1879 by de Bary as ‘the living together of unlike organisms’ – has a rich and convoluted history in biology. In part, because it questioned the concept of the individual, symbiosis fell largely outside mainstream science and has traditionally received less attention than other research disciplines. This is gradually changing. In nature organisms do not live in isolation but rather interact with, and are impacted by, diverse beings throughout their life histories. Symbiosis is now recognized as a central driver of evolution across the entire tree of life, including, for example, bacterial endosymbionts that provide insects with vital nutrients and the mitochondria that power our own cells. Symbioses between microbes and their multicellular hosts also underpin the ecological success of some of the most productive ecosystems on the planet, including hydrothermal vents and coral reefs. In November 2017, scientists working in fields spanning the life sciences came together at a Company of Biologists’ workshop to discuss the origin, maintenance, and long-term implications of symbiosis from the complementary perspectives of cell biology, ecology, evolution and genomics, taking into account both model and non-model organisms. Here, we provide a brief synthesis of the fruitful discussions that transpired.

## Abstract (precis)

**Summary:** At a recent Company of Biologists workshop, evolutionary biologists discussed the major outstanding questions in symbiosis research.

## Introduction

In recent years, symbiosis has gained recognition as one of the most important evolutionary processes shaping biodiversity throughout the history of life on Earth. Generally speaking, symbiosis refers to any type of intimate and long-term interaction between different organisms. A recent multidisciplinary workshop, supported by The Company of Biologists, entitled ‘Symbiosis in the microbial world: from ecology to genome evolution’ brought together researchers working at the forefront of the field to discuss symbioses involving the most numerically abundant and functionally diverse organisms on the planet, the microbes (which comprise bacteria, archaea and protists, as well as the viruses that infect them), and their interactions with multicellular hosts. These microbial symbioses range from metabolic (McCutcheon and Moran, 2012) and defensive interactions (Oliver et al., 2014) among free-living organisms, to the complete cellular and genomic integration that occurred during the endosymbiotic origins of mitochondria and chloroplasts in eukaryotic cells (Embley and Martin, 2006; Roger et al., 2017). Symbiosis provides an unparalleled route to evolutionary innovation, one that underlies some of the most important transitions in the history of life.

Owing to recent methodological breakthroughs, symbiosis research is undergoing a revolution. Characterising the genetic potential and metabolic capabilities of symbiotic partners has traditionally been challenging because most symbionts defy commonly applied enrichment and cultivation techniques. While many microbes are difficult to cultivate, symbionts may pose additional challenges because they often rely on interactions with other organisms in order to survive and, particularly in the case of endosymbionts, can rarely be cultivated on their own. However, the recent application of metagenomic and single-cell genomic approaches to the study of symbiosis now circumvents some of these issues by enabling the reconstruction of genomes from symbionts in their natural habitats (Siegl et al., 2011; Woyke et al., 2006). These techniques have greatly expanded our ability to sample existing symbiotic diversity and improved our understanding of interactions among and between microbes in the environment. This flood of new data has been complemented by proteomics (Mao and Franke, 2015), transfection and transformation systems enabling genetic manipulation of a wide range of organisms, and recent advances in experimental techniques such as single-cell imaging, microfluidics (Lambert et al., 2017), *in situ* hybridization, and secondary-ion mass-spectrometry (SIMS), allowing intracellular measurement of metabolic fluxes (Thompson et al., 2012). Collectively, these developments have opened up entirely new lines of symbiosis research, bringing both classical and emerging questions into the realm of tractable science.

Together with increased recognition of the fundamental importance of symbiosis to many areas of biology, this growth of activity is reflected in the rapidly expanding body of literature on the subject, including over 2500 publications in 2016 alone. Current research is proceeding on multiple fronts – from ecologists studying the diversity of microbial communities over large spatial scales to cell and evolutionary biologists investigating the long-term impacts of symbiosis on cell organization and genome evolution. The breadth of approaches and perspectives being brought to bear on symbiosis is a strength, but also represents a challenge as it involves researchers from different backgrounds who need to develop a shared language.

**Figure:**
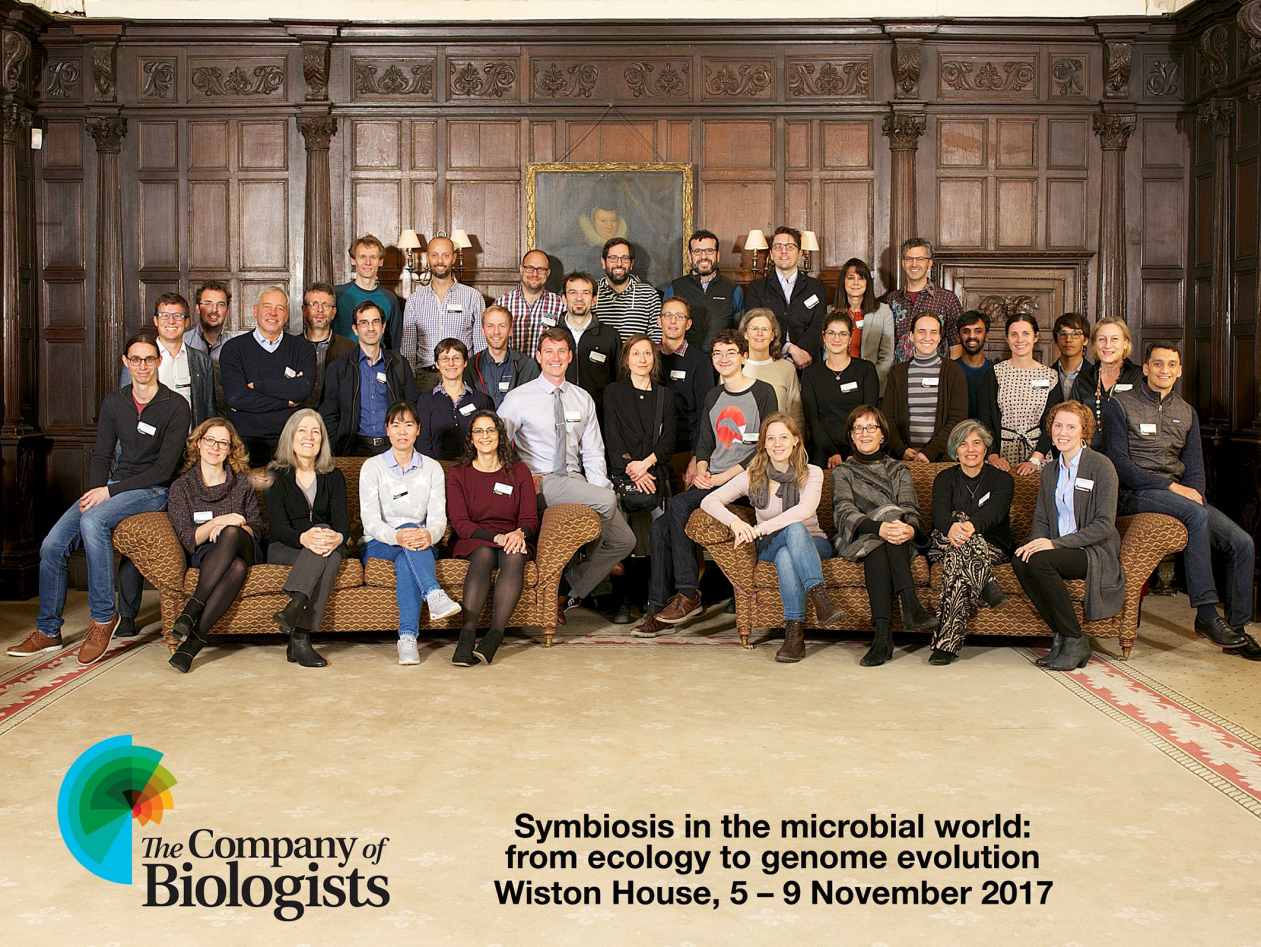

Workshop participants convened at Wiston House in Sussex, UK, with the aim of discussing the cellular, ecological, and evolutionary aspects of symbiosis, and its role in the history of life. Exciting new results on a broad range of symbiotic systems were presented, ranging from lab experiments on binary interactions between ciliates and their photosynthetic algal symbionts, to broad-scale analyses of complex microbial communities, such as those living in and on coral reefs. Collectively, these works employed a diversity of methodological approaches, including both traditional and cutting-edge cellular and molecular biology techniques, high-resolution imaging, molecular phylogenetics, and various ‘omics’ tools. The overall goal of the workshop was not only to stimulate fruitful discussions and to establish an integrative framework for research between all these fields, but also to identify the most important contemporary questions in the field of symbiosis research, questions that can only be tackled collaboratively by combining different tools, approaches and expertise. Here, we highlight points of consensus and controversy within and among these different fields and identify areas of opportunity for future multidisciplinary work.

### Symbiosis: what's in a name?

While the symbiosis research community is relatively small, its practitioners work in a variety of different areas and use diverse and often non-overlapping methodological approaches to explore a myriad of organismal associations, time scales and biological problems. Symbiotic associations span a gradient that includes mutualistic, commensal and even parasitic relationships. In addition, these associations can shift over ecological and evolutionary time and in response to changes in environmental conditions and community composition. Symbioses are often cast as facultative, ‘beneficial’ metabolic interactions between organisms that can evolve into obligatory interdependencies over time. Symbioses also vary in their level of cellular and genetic integration; they include ecto- and endosymbiotic interactions, in which an organism lives on the surface or within the cell(s) of another organism, respectively.

The most extreme cases of integration are the mitochondria and chloroplasts of eukaryotes, endosymbiotically-derived organelles that have long since lost their cellular autonomy (Archibald, 2015; Embley and Martin, 2006; Roger et al., 2017). At the other end of the spectrum are interactions between multicellular organisms and the microbes that live on and within them. The study of symbiosis leads to a broad range of questions, only some of which are easily applied to all systems. Indeed, given its tremendous scope, it is difficult to define what symbiosis is and what it is not. To what extent is the co-evolution between animals and their microbiomes symbiotic? Does the animal microbiome and its host represent a unit of selection and can/should it be considered a holobiont (Douglas and Werren, 2016; Skillings, 2016)? Which level of metabolic interaction and/or trophic relationship constitutes a symbiosis (Orphan, 2009; Schink, 2002)? When does an endosymbiont become an organelle (and how much does it matter) (McCutcheon and Keeling, 2014; Singer et al., 2017)? These are some of the questions that symbiosis researchers continue to grapple with.

### Reductionist and holistic approaches to symbiosis research

Some of the most spirited debates at the workshop centred on the scales at which questions about symbiosis can be most effectively addressed. These discussions were illustrative in that they made explicit certain differences in the accepted standards for evidence and methodological approaches between researchers working with tractable laboratory model systems on one hand, and those investigating the structure of complex natural communities on the other. Clearly, there are challenges in translating correlations and co-occurrence patterns reported in ecosystem and global-scale observational microbiome studies to specific, experimentally-tested functional interactions between partners. At the same time, we must also recognize that laboratory models do not necessarily fully capture the diversity and variability of symbiotic interactions that occur in nature, since the most tractable systems often involve few interacting partners.

Debates between reductionism and holism are common in science, but are particularly acute in symbiosis research because the strategy used often varies depending on the system being studied. Most accounts of the evolution of tightly-integrated, inter-dependent symbioses – as exemplified by the symbiotic bacteria of many insects (Moran et al., 2008) or the eukaryotic cell (Martin et al., 2015) – envisage an initial weak or transient interaction between the partners that evolves to become more stable and tightly integrated over time through neutral and/or adaptive processes (Lukeš et al., 2011; Szathmáry, 2015; Timmis et al., 2004; West et al., 2015). If this scenario is generally correct, then holistic and reductionist approaches are perhaps best suited to studying different ends of the symbiotic continuum, from a complex mix of mostly transiently interacting organisms to a much smaller set of tightly integrated partners. Top-down and bottom-up approaches to symbiosis research can be complementary: experimental work on lab models is clearly essential for providing fundamental mechanistic insight into how symbiosis works. At the same time, observational and whole-community analyses can generate hypotheses to be tested with established models, and can also suggest which new model systems need to be brought into the lab – an expensive and time-consuming process – in order to address the major outstanding questions.

### Microbial community stability over space and time

Another nascent dimension of symbiosis research is the focus on understanding the evolutionary and ecological processes that drive the changes in patterns of symbiosis observed over short and long time scales. The first challenge is to determine how stable symbiotic microbial communities are over time and how much can be generalized from a small number of observations of natural systems that are not easily tamed in the lab. However, the study of community composition over time has revealed that some systems show high levels of variability while others are extremely stable. In order to understand these patterns, it becomes imperative to not only take into account the high-level taxonomic diversity that comprises ecological communities, but also the functional traits that are associated with each taxon. It might, therefore, also be important to model symbiont systems based on their functional traits in addition to their taxonomic composition, because cases have been described in which the former remains stable, while the latter appears to vary (Lozupone et al., 2012), at least at certain levels of functional and taxonomic granularity [Douglas has referred to this as the ‘inconstant microbiome’ (Wong et al., 2013)]. Seen from this perspective, perhaps the most relevant unit of selection is the metabolic function performed by the interacting unit, given that similar processes can be performed by taxa (or genes) that are only distantly related: according to Doolittle and Booth, ‘it's the song, not the singer’ (Doolittle and Booth, 2017).

### The meta-omics black box: from data to biology

The inferences derived from high-throughput analyses of environmental DNA, protein sequences and/or chemical compounds are only as strong as the databases used to annotate them, and a major current roadblock is the prevalence of genes, proteins and molecules with no known function. A recent effort to define the minimal genome required for a self-replicating bacterium provided a humbling perspective: out of the 473 genes in *Mycobacterium* supporting a viable and free-living cell, 32% have an unknown function (Hutchison et al., 2016). This highlights our very incomplete understanding of the molecular basis of vital biological processes. Currently, 50-80% of meta-omics data in hand cannot be annotated, which leads to an incomplete picture of the systems being studied by restricting the interpretation of the results to biochemical pathways and cellular processes that are already well understood. This issue is frequently encountered in symbiotic systems, which can be reservoirs of novel accessory genes due to niche-specificity (Porter et al., 2016; Remigi et al., 2016) and the lack of cultivated representatives of numerous microbial partners. Such genes might play particularly important roles in symbiotic relationships and could thus represent important targets for future studies – if only they can be identified. Unassigned data should therefore not be dismissed, but we should instead encourage the development and use of novel analytical tools capable of delving into their coding potential and putative functions (Hartmann et al., 2017). Furthermore, it is important to keep in mind that functions inferred for proteins with homologous sequences in current databases cannot be fully relied upon. For instance, even if general enzymatic properties are conserved, substrate specificity and/or reaction directionality can often not be predicted based on homology alone (Laso-Pérez et al., 2016). Therefore, hypotheses on the biology of host-symbiont interactions based solely on genomic data should ideally be experimentally validated.

### Future directions in symbiosis research

Many ecologically important host-symbiont systems cannot be easily cultivated or genetically manipulated. However, microbial isolation is returning to the spotlight and the coming years are likely to see new advances in axenic culturing techniques (Overmann et al., 2017). The semantic shift from ‘unculturable’ to ‘not yet cultivated’ is a very encouraging sign and some microbes long thought to be obligate intracellular symbionts are now grown axenically (Omsland et al., 2013). Metabolic pathway reconstruction of uncultured bacteria can already be used to predict their nutrient requirements and rationally design new culture conditions. In the near future, this will enable us to not only get a better understanding of the biology of organisms involved in symbiosis, but also to genetically manipulate them, which will in turn lead to greater insights into the mechanisms that regulate symbiotic interactions and host colonization. Other avenues for future research should also include the development of techniques to identify bacterial symbionts in natural communities (Orphan, 2009). This could be achievable by identifying phenotypic or genomic traits that are predictive of symbiotic interactions (Moran and Wernegreen, 2000) and might help to decipher how symbionts are acquired or transmitted.

In addition to these technical developments, significant efforts should be made to generate high-quality reference genomes from single-celled eukaryotes, which comprise most of eukaryotic diversity. We will need such data in order to make proper sense of metagenomic and metatranscriptomic datasets generated from diverse environments, as well as to fully grasp the diversity of symbiotic relationships in nature (Sibbald and Archibald, 2017). Over deeper evolutionary timescales, there is still much to learn about how and when the mitochondrial endosymbiosis occurred and its role in the origin of the eukaryotic cell (Eme et al., 2017; Roger et al. 2017). Future sequencing and cultivation efforts will hopefully allow us to identify and study close relatives of the elusive prokaryotic ancestors of eukaryotes (Spang et al., 2015; Zaremba-Niedzwiedzka et al., 2017), thereby allowing us to refine hypotheses on the origin of the eukaryotic cell (Eme et al., 2017).

Given the breadth and novelty of the work presented at the workshop, the future is undoubtedly bright for symbiosis research. Methodological advances combined with efforts to further stimulate multidisciplinary approaches will inevitably provide profound insights into microbial symbioses and unveil fundamental aspects of the complex interactions that characterize life on Earth.
